# Diagnostic accuracy of a short-duration 3 Tesla magnetic resonance protocol for diagnosing stifle joint lesions in dogs with non-traumatic cranial cruciate ligament rupture

**DOI:** 10.1186/1746-6148-9-40

**Published:** 2013-02-28

**Authors:** Vladimir Galindo-Zamora, Peter Dziallas, Davina C Ludwig, Ingo Nolte, Patrick Wefstaedt

**Affiliations:** 1 Small Animal Hospital, University of Veterinary Medicine Hannover, Foundation. Bünteweg 9, D-30559, Hannover, Germany; 2 Small Animal Clinic, Faculty of Veterinary Medicine, National University of Colombia, Carrera 30 # 45-03 (Ciudad Universitaria), Bogotá, Colombia

**Keywords:** Dog, Stifle, Cranial cruciate ligament, High-field MRI, Radiography

## Abstract

**Background:**

Magnetic resonance (MR) imaging is the preferred diagnostic tool to evaluate internal disorders of many joints in humans; however, the usefulness of MR imaging in the context of osteoarthritis, and joint disease in general, has yet to be characterized in veterinary medicine. The objective of this study was to assess the diagnostic accuracy of short-duration 3 Tesla MR imaging for the evaluation of cranial and caudal cruciate ligament, meniscal and cartilage damage, as well as the degree of osteoarthritis, in dogs affected by non-traumatic, naturally-occurring cranial cruciate ligament rupture (CCLR). Diagnoses made from MR images were compared to those made during surgical exploration. Twenty-one client-owned dogs were included in this study, and one experienced evaluator assessed all images.

**Results:**

All cranial cruciate ligaments were correctly identified as ruptured. With one exception, all caudal cruciate ligaments were correctly identified as intact. High sensitivities and specificities were obtained when diagnosing meniscal rupture. MR images revealed additional subclinical lesions in both the cranial and caudal cruciate ligaments and in the menisci. There was a “clear” statistical (kappa) agreement between the MR and the surgical findings for both cartilage damage and degree of osteoarthritis. However, the large 95% confidence intervals indicated that evaluation of cartilage damage and of degree of osteoarthritis is not clinically satisfactory.

**Conclusions:**

The presence of cruciate ligament damage and meniscal tears could be accurately assessed using the MR images obtained with our protocol. However, in the case of meniscal evaluation, occasional misdiagnosis did occur. The presence of cartilage damage and the degree of osteoarthritis could not be properly evaluated.

## Background

The pathologic changes most commonly seen in the stifle joints of dogs suffering from cranial cruciate ligament rupture (CCLR) are osteoarthritis, osteophytosis and meniscal tears [1,2]. In humans, magnetic resonance (MR) imaging is the preferred diagnostic method for assessment of periarticular soft tissue and articular cartilage, including evaluation of lesions such as meniscus and ligament tears in the knee [2-6]. However, the usefulness of MR for the assessment of osteoarthritis, and joint disease in general, is still not well characterized in veterinary medicine [6].

Low-field (LF) MR imaging has been found useful for the evaluation of both normal and diseased stifle joints in dogs [7-9]. One study found 0.3 Tesla (T) MR imaging helpful for the diagnosis of complete tears in the canine meniscus when compared with arthroscopy, especially in larger dogs [10]. However, another study, which compared LF MR imaging with arthroscopy, found that LF MR imaging (0.5 T) failed to identify 44% of meniscal tears found during arthroscopy [2].

The introduction of high-field (HF) MR magnets has significantly improved MR image quality and the accuracy of assessment of subchondral bone lesions, joint spaces, soft tissues, cartilage defects and osteophyte growth in canine stifles with experimentally-induced osteoarthritis [1,11]. One study, which compared the use of 1.5 T MR with computed radiography to assess osteophytosis, subchondral bone sclerosis, joint effusion and soft tissue thickening after experimentally-induced osteoarthritis in dogs, found that MR was more sensitive than radiography for detection of the onset and progression of osteophytosis [1]. Another study found that 1.5 T MR detected meniscal tears in clinical cases of CCLR with a sensitivity of 100% and a specificity of 94% [3].

However, to the authors’ knowledge there are no previously published studies evaluating 3 T HF MR imaging in clinical cases of non-traumatic canine stifle pathology. Accurate MR-based diagnosis of joint pathology could prevent unnecessary surgical exploration during procedures such as tibial plateau leveling osteotomy (TPLO) or tibial tuberosity advancement (TTA), which do not themselves require opening the joint. In addition, a short-duration MR protocol would reduce total examination time, and therefore also reduce anesthetic time (and risk).

The objective of this study was to assess the diagnostic accuracy of images obtained by 3 T MR scan using a short-duration protocol for determination of joint lesions in dogs. MR findings were compared with actual surgical findings in dogs with surgically-confirmed CCLR. We hypothesized that the images obtained using this MR protocol would allow accurate, non-invasive diagnosis of morphological changes within the canine stifle, which would highly correlate with the surgical findings while keeping examination times short.

## Methods

The study was carried out in accordance with the animal welfare guidelines of the State of Lower Saxony. No ethical approval was obtained, as the MR examinations were part of the diagnostic database of each patient; however, all owners agreed to their pet’s participation in the study and signed a consent form.

### Patients

Twenty-one adult dogs that presented to the Small Animal Hospital of the University of Veterinary Medicine Hannover, Foundation (Germany), with non-traumatic hind limb lameness originating from the stifle joint were included in the study. CCLR was suspected based on stifle pain or inflammation, a positive cranial drawer test, a positive tibial compression test and/or compatible radiographic changes in the stifle. CCLR was confirmed in all cases by exploratory arthrotomy at the time of the surgical treatment. The MR images of three clinically and radiographically healthy stifles were also included for evaluation to reduce evaluator bias.

### Procedures

Physical status was determined based on clinical examination, blood work and other diagnostic tests as needed. Based on the American Society of Anesthesiologists (ASA) physical status classification system, all patients were classified as ASA 2 (patients with local or mild systemic disease). Thus, the dogs were anesthetized using a combination of acepromazine^a^ (0.05 mg/kg), levomethadone^b^ (0.6 mg/kg), propofol^c^ (1–5 mg/kg) and isoflurane^d^ in oxygen (1–2.5%); for additional intra- and post-operative analgesia carprofen^e^ (4 mg/kg) was given.

Once under anesthesia, the animals were moved to the MR suite and positioned in lateral recumbency with the limb to be examined in a non-dependent position and the stifle joint at an angle of ~135°. Using a state-of-the-art 3 T MR scanner^f^, images were obtained from the affected stifle. Small (11 cm Ø) surface ring coils^g^ were used as image enhancers; these were positioned parallel to each other, lateral and medial to the affected stifle, with the joint centered between the two coils. The MR protocol included: a 3-D (3-dimensional) PDW (proton-density weighted) acquisition sequence, which was afterwards reconstructed in sagittal, dorsal and transverse planes; a PDW HR (high-resolution) TSE (turbo spin echo) SENSE (sensitivity encoding for fast MR) sequence in sagittal and dorsal planes; a PDW HR SPAIR (spectrally adiabatic inversion recovery) SENSE in the sagittal plane; and a T1-weighted TSE clear (constant level appearance) sequence in the sagittal plane (Table 1). This protocol had been previously standardized and is regarded as suitable for use in clinical cases, since diagnostic image quality is optimal and acquisition time is only 22 minutes (total examination time is about 40 minutes including positioning, reference scan, survey, and sequence planning).

**Table 1:** Magnetic resonance imaging sequence parameters used in this study

**Sequence**	**Plane**	**TR**	**TE**	**Slice (mm)**	**Gap (mm)**	**FOV (mm)**	**Flip angle**	**Matrix**	**Orientation**
PDW	3-D	1300	34			100 × 100 × 70		220 × 167	Joint centered
PDW	Sagittal			2			90°		True sagittal
PDW	Dorsal			2			90°		Parallel to patellar ligament
PDW	Transverse			2			90°		Parallel to tibial plateau
PDW HR aTSE SENSE	Sagittal	4326	30	2	0.2	120 × 120 × 48	90°	480 × 296	True sagittal
PDW HR aTSE SENSE	Dorsal	4324	30	2	0.2	120 × 120 × 48	90°	344 × 235	Parallel to patellar ligament
PDW HR SPAIR SENSE	Sagittal	4701	30	2	0.2	800 × 800 × 46	90°	228 × 160	True sagittal
T1-weighted TSE clear	Sagittal	665	18	1.8	0.18	90 × 90 × 39	90°	180 × 134	True sagittal

*TR* = Repetition time; *TE* = Echo Time; *FOV* = Field of view; *PDW* = proton-density weighted; *3-D* = 3-dimensional; *HR* = high resolution; *TSE* = turbo spin echo; *SENSE*: sensitivity encoding; *SPAIR* = spectrally adiabatic inversion recovery; clear = constant level appearance.

**Figure 1:**
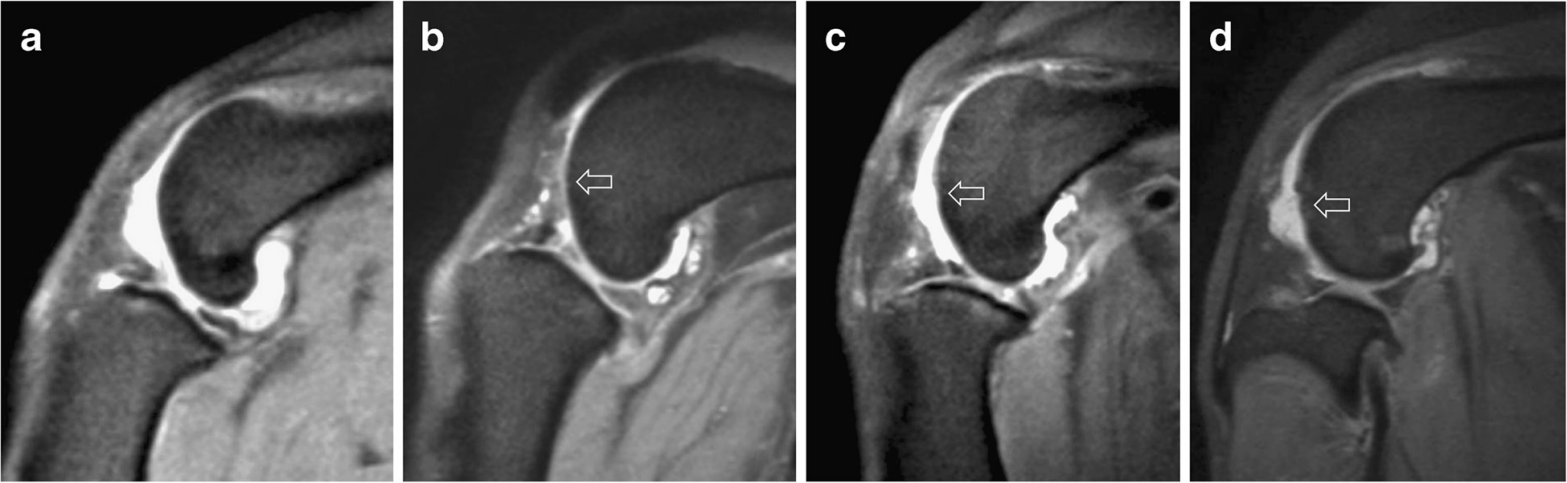
**Examples of PDW images (sagittal plane) in which the degree of cartilage damage was correctly graded using the scoring system shown in Table**2**.** The arrows indicate representative cartilage lesions. **a** (Score 0): Smooth normal cartilage; **b** (Score 1): Mild irregularities of the cartilage surface; **c** (Score 2): Partial thickness erosion; **d** (Score 3): Ulceration with exposure of subchondral bone.

**Figure 2:**
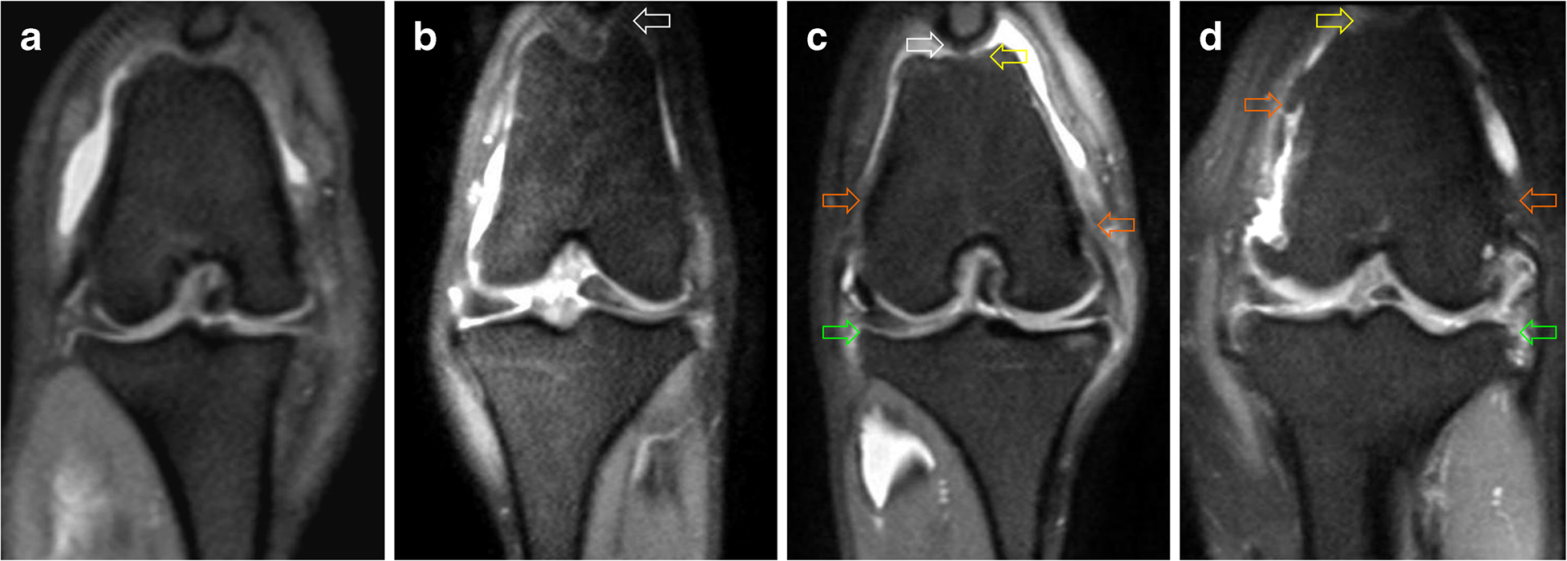
**Examples of PDW images (dorsal plane) illustrating the osteoarthritis scoring system.** The images belong to patients correctly graded according to the scoring system described in Table 2. **a** (Score 0): Osteophytes absent. **b** (Score 1): Osteophytes present on the patella (not shown) and proximal aspect of the femoral trochlear groove (arrow). **c** (Score 2): Osteophytes present on the patella (white arrow), femoral trochlear groove (yellow arrow), medial and lateral femoral condyles (orange arrows) and tibial plateau (green arrow). **d** (Score 3): Severe osteophytes on the patella (not shown), femoral trochlear groove (yellow arrow), medial and lateral femoral condyles (orange arrows) and tibial plateau (green arrow).

In the MR images, the signal intensities of the cranial and caudal cruciate ligament (CdCL) were evaluated. Changes in signal intensity and evidence of meniscal rupture, defined as a linear increase in intrameniscal signal intensity penetrating a meniscal surface or the presence of complex signal changes or meniscal distortion (representing longitudinal or bucket handle tears) [10], were also recorded. The degree of cartilage damage and osteoarthritis were assessed using a previously published scoring system (Table 2, Figures 1 and 2) [6,12].

**Table 2:** The scoring system used to grade osteoarthritis and cartilage damage in this study

**Parameter**	**Scoring system**	**MRI**	**Surgery**
Cartilage damage*	Smooth normal cartilage	0	0
	Mild surface irregularities	1	1
	Partial thickness erosion	2	2
	Ulceration with exposure of subchondral bone	3	3
Osteoarthritis**	Osteophytes absent	0	0
	Osteophytes present on patella and proximal aspect of femoral trochlear groove	1	1
	Osteophytes present on patella, femoral trochlear groove, medial and lateral	2	2
	femoral condyles and tibial plateau		
	Severe osteophytes on patella, femoral trochlear groove, medial and lateral	3	3
	femoral condyles and tibial plateau		

*MRI* = Magnetic resonance imaging; * Adapted from Olive et al. 2010; ** Adapted from Moreau et al. 2003.

To avoid under- or over-interpretation of the findings, a set of images was prepared exemplifying each score for the evaluated parameters. Examples from healthy joints were obtained from patients undergoing MR examination as part of another study (not yet published); examples from diseased joints were obtained from previous patients admitted to the hospital, whose lesions were surgically confirmed, as well as from some of the patients enrolled in this study. The use of these example images has been previously reported to increase the accuracy of studies similar to this one [6]. MR images of all patients were evaluated by an experienced veterinarian (VGZ) blinded to the clinical and surgical findings of each patient. He initially became familiar with the reference images, and then proceeded to evaluate MR images of the patients. All images were analyzed using a high-resolution diagnostic screen^h^.

After the MR scan, the patients were moved to the operating room for treatment. A lateral parapatellar arthrotomy was performed and the joint was explored. The surgical examination was considered the gold standard for evaluation of all parameters. The surgeons examined the cranial and caudal cruciate ligaments and determined their integrity. The menisci were exposed by displacing the tibia cranially using a Hohmann elevator, and the presence of gross macroscopic changes (e.g. distortion or a bucket handle tears) was determined. Additionally, the menisci were palpated using an exploration probe, to detect the presence of tears. The lateral and medial articular surfaces of the femur and the articular surface of the patella were exposed using a Langenbeck retractor and photographed. The same evaluator scored the degree of cartilage damage and osteoarthritis on all photographs. Finally, joints were treated intra-operatively as required based on each individual patient’s pathologic changes, and a previously described extracapsular technique [13] was used to stabilize them. Postoperative recovery was uneventful in all cases.

### Statistical analyses

The sensitivity and specificity of MR imaging for the diagnosis of lateral and medial meniscal tears were calculated (including 95% confidence intervals). A Fisher’s Exact Test was performed to determine the association between rows and columns and was considered statistically significant if *p* < 0.05. A Cohen’s kappa coefficient (κ) was used to assess the statistical agreement between the parameters cartilage damage and osteoarthritis, and the surgical findings. Since these parameters had more than 2 scoring options, the weighted κ value was used for statistical analysis as it takes into account the extent to which the evaluations disagree [14]. 95% confidence intervals were also calculated. Statistical agreements were described as “no agreement” (<0.1000), “weak” (0.1000–0.4000), “clear” (0.4100–0.6000), “strong” (0.6100–0.8000) and “almost perfect” (0.8100–1.0000) [15].

Sensitivities and specificities (including their confidence intervals) as well as the Fisher’s Exact Test were calculated using GraphPad Prism® Version 4 (GraphPad Software, Inc. La Jolla, CA, USA). κ calculations were performed using SAS for Windows® SAS 9.2 TS Level 1 M0 (SAS Institute Inc, Cary, NC, USA).

## Results

### Patients

Eight mixed-breed dogs, four Labrador Retrievers, two Beagles, and one German Shepherd, Great Dane, Boxer, Griffon, Bernese Mountain Dog, Rottweiler and Small Munsterlander were included in this study. There were eleven females and ten males, with a mean age of 5.71 years (range 2–11 years) and mean weight of 31.6 kg (range 7–56 kg). Twelve left stifles and 9 right stifles were affected.

### Evaluation of stifle joint pathology

All three normal stifle joints were classified as such and no affected joint was misclassified as normal during blinded evaluation of MR images.

#### Cruciate ligaments

**Figure 3:**
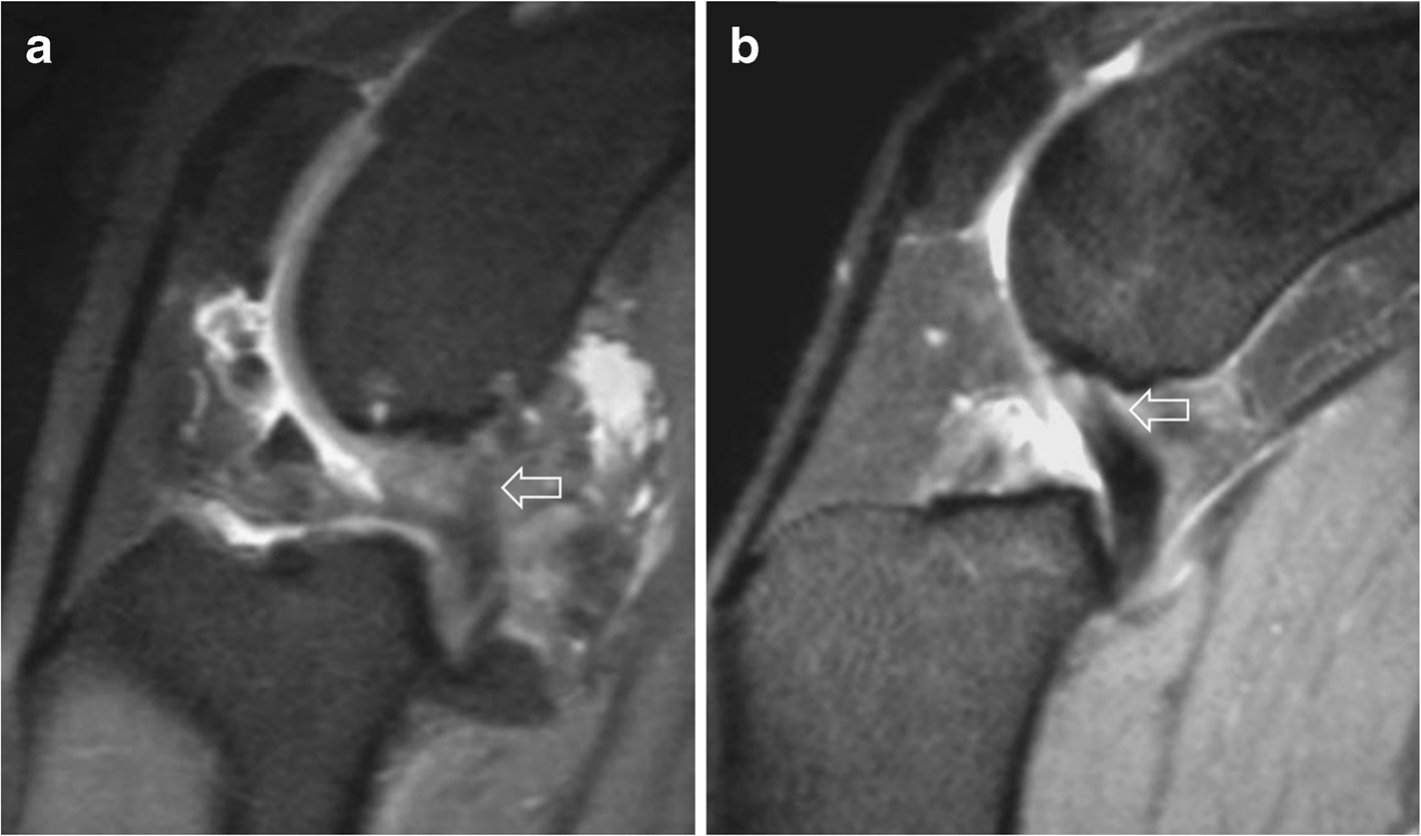
**Examples of PDW images (sagittal plane) of caudal cruciate ligament abnormalities. a**: Abnormal appearance (generalized hyperintensity) of the caudal cruciate ligament (arrow) seen in one patient (see text). **b**: Areas of hyperintensity (arrow) within the caudal cruciate ligament as observed in several patients. In both images the cranial cruciate ligament cannot be seen.

CCLR was confirmed during surgery in all 21 cases. Of the 21 cases, there were 18 complete and 3 partial ruptures. However, increased signal intensity in MR images led to an incorrect diagnosis of partial rupture based on MR images in two cases, even though the ligaments were found to be completely torn during surgical exploration. In another three cases, the evaluator observed a complete loss of fiber continuity in the MR images leading to a diagnosis of a complete rupture of the CrCL, but only a partial rupture was present at surgery. In all other cases (16) a correct diagnosis of total rupture was made based on MR images. All CdCLs except one were correctly identified as non-ruptured on the MR images, the one exception was diagnosed as partially torn (Figure 3a); during surgery it was observed that this particular patient had an abnormally thin (degenerated) CdCL. In spite of the fact that none of the other CdCLs were ruptured, another 13 patients (61.9%) had areas of hyperintensity within the CdCL (Figure 3b) in the MR images; these were interpreted as subclinical changes and were not detectable during surgery. The remaining CdCLs showed no changes.

#### Menisci

**Figure 4:**
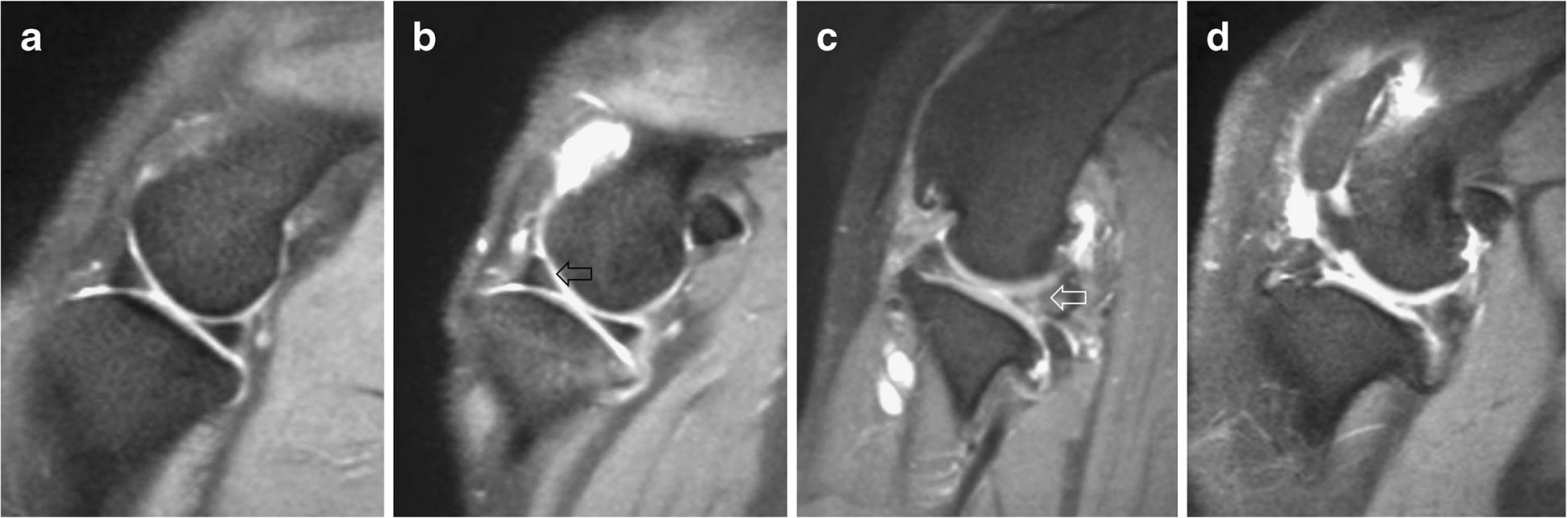
**Examples of PDW images (sagittal plane) of the menisci. a**: A normal medial meniscus. **b**: Hyperintensity in the cranial horn of the lateral meniscus (arrow). **c**: Rupture of the caudal horn of the lateral meniscus (note the presence of increased intrameniscal signal intensity penetrating the articular meniscal surface [arrow]). **d**: Rupture of both the caudal and cranial horn of the medial meniscus (note the presence of complex signal changes [caudal horn] and meniscal distortion [cranial horn]).

The meniscal lesions found by MR examination are compared with those seen during surgery in Table 3, and example images are shown in Figure 4. It is important to clarify that in the MR images, areas of hyperintensity (H) indicate meniscal changes but not rupture. As seen in Table 3, medial meniscal tears were found during surgery in 18 patients (86.71%). Only one of these patients also had a tear of the lateral meniscus. The remaining three patients had no meniscal pathology at surgery. The sensitivity and specificity of MR imaging for the diagnosis of meniscal tears were analyzed for each meniscus and are summarized in Table 4.

**Table 3:** Meniscal lesions found by magnetic resonance (MR) examination and during surgery

	**Lateral meniscus**			**Medial meniscus**	
	MR findings*	**Surgical findings**		MR findings*	**Surgical findings**
**Patient**		**Rupture**	**Patient**		**Rupture**
1	H	No	1	R	Yes
2	H	No	2	R	Yes
3	0	No	3	R	Yes
4	0	No	4	R	Yes
5	H	No	5	0	Yes
6	0	No	6	R	Yes
7	R	Yes	7	R	Yes
8	0	No	8	0	No
9	0	No	9	R	Yes
10	0	No	10	R	Yes
11	0	No	11	R	Yes
12	0	No	12	R	Yes
13	0	No	13	0	No
14	0	No	14	R	Yes
15	0	No	15	R	Yes
16	H	No	16	0	No
17	H	No	17	R	Yes
18	0	No	18	R	Yes
19	0	No	19	R	Yes
20	0	No	20	R	Yes
21	H	No	21	R	Yes

* Adapted from Martig et al. 2006; *H* = Hyperintensity (area[s] of intrameniscal increase of signal intensity that do not reach any surface); *R* = Rupture (a linear increase in intrameniscal signal intensity that penetrates the meniscal surface, complex signal changes or meniscal distortion); *0* = Low and homogeneous signal intensity.

**Table 4:** Sensitivity and specificity of short-duration 3 T magnetic resonance (MR) imaging for the evaluation of meniscal rupture

**Lateral meniscus**		Surgery (rupture)	Surgery (no rupture)
	MR (rupture)	1	0
	MR (no rupture)	0	20
	Sensitivity (95% CI):	1.0000 (0.0250–1.0000)	
	Specificity (95% CI):	1.0000 (0.8317–1.0000)	
	F:	0.0476	
**Medial meniscus**		Surgery (rupture)	Surgery (no rupture)
	MR (rupture)	17	0
	MR (no rupture)	1	3
	Sensitivity (95% CI):	0.9444 (0.7270–0.9986)	
	Specificity (95% CI):	1.0000 (0.2924–1.0000)	
	F:	0.0030	

*CI* = Confidence interval; *F* = Fisher’s exact test p value (statistically significant if p ≤ 0.05).

#### Cartilage damage and degree of osteoarthritis

The κ values (including 95% confidence intervals) for the degree of cartilage damage and degree of osteoarthritis were 0.4118 (0.1730–0.6506) and 0.5333 (0.3081–0.7586), respectively. Both agreements were statistically defined as “clear” (see statistical analyses section).

## Discussion

This study reports the diagnostic accuracy of a short-duration, 3 T MR imaging protocol to detect damage to the cruciate ligaments, menisci and cartilage of the stifle joint, as well as the degree of osteoarthritis present, in dogs with non-traumatic rupture of the CrCL. Diagnoses based on MR images were compared with surgical findings, which were considered the gold standard. All patients were adult dogs with non-traumatic rupture of the CrCL. The majority were mixed-breed dogs and Labrador Retrievers, this is similar to the breed profile of dogs with CCLR reported in a previous study [16].

All MR images came from diseased stifles and the evaluator was aware of this; therefore, images of healthy joints were included in an attempt to improve the accuracy of the study and reduce bias. However, it has been previously reported that healthy joints are easily recognized in MR images [3]. The MR diagnosis of a total CCLR is also relatively straightforward, since the loss of fiber continuity is readily seen on the images (Figure 3). A partial CCLR is more difficult to diagnose because the only change observed is an increased signal intensity in the normally hypointense ligament [17]. Nevertheless, this is not clinically important, since both total and partial ruptures lead to pathologic changes [18], and medical or surgical therapy is indicated.

The misdiagnosing of one intact CdCL as partially ruptured on the MR images was the result of severe degenerative changes within the ligament that resulted in macroscopic thinning that was visible during surgery. This particular patient also had severe joint changes, including ruptures of both menisci, osteoarthritis (score 3), cartilage damage (score 3) and generalized thickening of the joint capsule. Thus, the abnormal ligament was one part of a whole organ (joint) disease state. Areas of hyperintensity in the CdCL visible on MR images very likely represent subclinical changes. Previous reports of naturally-occurring CdCL damage are scarce, and none of them deal with MR imaging [19,20]. However, a study performed in medium to large breed dogs suffering from CCLR, in which the CdCL was directly visualized (by arthroscopy or arthrotomy) at the time of surgical stabilization, reported that 88% of the patients showed some degree of damage to the CdCL [21]. Additionally, a previous experimental study described significant extracellular matrix disorganization (degenerative changes and change in the collagen fibril diameter pattern) in the CdCL of CrCL-deficient dogs 2 years after ligament transection [22]. It is possible that, in the initial phases of the degeneration process, an inflammatory phase takes place leading to the areas of hyperintensity seen in this study. This has been described for the CrCL [17], and it is logical to assume that similar changes can occur in the CdCL. These lesions are not a surprising finding as it has been shown that the consequences of experimental transection of the CrCL include strong torsion and tensile stresses on the caudal ligamentous structures of the stifle [23]. The clinical significance of these changes in the CdCL is yet to be determined; however, we suggest that one reason for the persistent lameness occasionally seen after surgical stabilization might be the changes in the CdCL.

Meniscal damage has been reported in as many as 80% of the dogs suffering from CCLR [24], with the medial meniscus most commonly affected [16]. In our study, the incidence of torn medial menisci was even higher (86.71%). Although meniscal lesions are more common in patients suffering from chronic CCLR [18], in this study we found meniscal tears not only in patients with chronic lameness, but also in patients with only a 2-week history of lameness. The reason for this high incidence of meniscal damage is unclear. There were also many subclinical meniscal lesions (areas of hyperintensity) visible on MR images. These lesions were not detected at surgery, as they were either the result of subclinical, degenerative changes or were located in non-visible areas of the meniscus; both instances have been previously reported [5,25]. It is also possible that those subclinical lesions were in fact tears not detected during surgical exploration: a previous report found that arthrotomy had a lower sensitivity, specificity and correct classification rate, in comparison to arthroscopy, when examining meniscal tears [26]. Thus, arthrotomy might have not been the ideal method for assessing the menisci. However, in the aforementioned study only the medial meniscus was evaluated, whereas the findings classified as subclinical lesions in our study were seen on the lateral meniscus, which is relatively easy to observe during lateral arthrotomy. In any case, these findings might be of prognostic value, since these menisci may have an increased risk of rupture after surgery. This likelihood could have been assessed by performing a follow-up evaluation of our patients, but that was beyond the scope of this study.

The diagnostic accuracy of MR examination for detecting meniscal tears was very satisfactory. In the case of lateral meniscal rupture, the sensitivity and specificity of MR were very high; however, since there was only one case of lateral meniscal rupture, the sensitivity showed a very wide confidence interval (Table 4). For the detection of tears of the medial meniscus, MR had a higher and more reliable specificity than sensitivity, this may be due to the high number of ruptured medial menisci (Table 4). However, the confidence intervals for the specificity of MR for tear detection indicate that MR was less specific in the medial meniscus than in the lateral meniscus. A previous review of 59 human studies, which evaluated 7367 MR scans and 5416 arthroscopies, also found a lower specificity in the medial meniscus in comparison to the lateral one [4]. In the veterinary literature, previous reports on the utility of MR for detection of meniscal injury are mixed. One previous study did not find LF MR imaging useful for diagnosing meniscal tears in dogs [2]. However, another study using HF MR imaging (1.5 T) found a global sensitivity of 100% and a specificity of 94% for the diagnosis of meniscal tears using PD TSE sequences [3]. These sequences are similar to those used in our study, suggesting that our results are reliable, especially considering the stronger magnetic field of our scan. If we keep in mind that any diagnostic method used to diagnose a meniscal rupture should be highly sensitive, with a reasonably high specificity [2], the results obtained with the short-duration protocol used in the present study are encouraging. Additionally, the F values (indicating row/column associations) for both menisci are statistically significant (Table 4). In spite of this, some patients can still be misdiagnosed as having a tear, and some can be misdiagnosed as having intact menisci. Hence, it seems evident that accurate interpretation of meniscal damage from MR images is challenging, and that drawing the line between subclinical and clinically relevant changes is a difficult task.

The results of the cartilage damage and osteoarthritis scoring were less than satisfactory. Statistical agreements between scores based on MR images and scores based on visualization during surgery were “clear”, but the 95% confidence intervals were very wide. Therefore, the evaluation of cartilage damage and degree of osteoarthritis using MR is not clinically reliable. The results of the cartilage scoring agree with previous human studies, which have shown that MR cannot replace direct visualization for diagnosing cartilage damage in the knee [27,28]. However, a recent study performed to evaluate the metacarpophalangeal articular cartilage in horses demonstrated that it is possible to satisfactorily evaluate cartilage thickness and structure using a fat-suppressed spoiled gradient-recalled imaging technique [29]. Thus, perhaps a small increase in acquisition times and/or the use of other sequences could improve the diagnostic accuracy of cartilage damage grading in dogs. Since one important goal of the present study was to keep examination times to a minimum, we did not attempt to use additional protocols in this study. The results of our osteoarthritis scoring indicate that 3 T MR imaging is not particularly reliable for the scoring of osteoarthritis.

### Limitations

Our study provides new information; however, there were limitations. The most important limitation was that only one evaluator assessed the images, and that he was aware that the dogs had a clinical diagnosis of CCLR. This could have biased the results. The small sample size is another important limitation. Finally, even though the evaluator was experienced in reading MR images of canine stifles, the lack of a board-certified radiologist for interpretation of the images may have also been a limitation of this study.

## Conclusions

In this study, the usefulness of a short-duration, 3 T MR protocol for assessing joint pathology associated with non-traumatic CCLR was evaluated. Parameters such as cruciate ligament rupture and the presence of meniscal lesions could be properly assessed. However, in the case of meniscal evaluation some margin for misdiagnosis is present. Furthermore, cartilage damage and osteoarthritis scoring based on MR images was not satisfactorily accurate. Thus, the MR protocol still needs to be improved. In spite of this, it was remarkable that image quality allowed a relatively accurate diagnosis of the most clinically relevant parameters, even in the smallest patient (7 kg). The correct diagnosis of meniscal lesions may prevent the surgeon from performing unnecessary stifle joint explorations.

Future studies could focus on standardizing sequences that may improve image quality of all stifle structures while keeping the examination times to a minimum.

## Endnotes

^a^Vetranquil® 1%: Albrecht GmbH, Aulendorf, Germany.

^b^L-Polamivet®: Intervet Deutschland GmbH, Unterschleißheim, Germany.

^c^Narcofol® 10 mg/ml: CP-Pharma Handelsgesellschaft GmbH, Burgdorf, Germany.

^d^Isofluran CP®: CP-Pharma Handelsgesellschaft GmbH, Burgdorf, Germany.

^e^Rimadyl® Injektionslösung: Pfizer GmbH, Berlin, Germany.

^f^Philips Achieva 3.0 T X-series MRI. Philips Healthcare, Hamburg, Germany.

^g^Achieva 3.0 T Musculoskeletal SENSE Flex S coil 2 elements.

^h^http://EIZO RadiForce™ RX211 Medical color LCD monitor. Enzo Nanao Corporation, Hakusan, Ishikawa, Japan.

## Competing interests

The authors declare that they have no competing interests.

## Authors’ contributions

VGZ participated in study design, developed the scoring system, performed data collection and analysis, read the images and wrote the manuscript. PD and DCL performed the MRs. IN had the original conception of the project, participated in study design, performed the arthrotomies and approved the final version of the research project. PW designed, coordinated, approved and supervised all aspects of the study. All authors have critically revised the manuscript and read and approved the final manuscript.
